# Hamburger polyomaviruses

**DOI:** 10.1099/vir.0.000033

**Published:** 2015-04

**Authors:** Alberto Peretti, Peter C. FitzGerald, Valery Bliskovsky, Christopher B. Buck, Diana V. Pastrana

**Affiliations:** National Cancer Institute, Bethesda, MD, USA

## Abstract

Epidemiological studies have suggested that consumption of beef may correlate with an increased risk of colorectal cancer. One hypothesis to explain this proposed link might be the presence of a carcinogenic infectious agent capable of withstanding cooking. Polyomaviruses are a ubiquitous family of thermostable non-enveloped DNA viruses that are known to be carcinogenic. Using virion enrichment, rolling circle amplification (RCA) and next-generation sequencing, we searched for polyomaviruses in meat samples purchased from several supermarkets. Ground beef samples were found to contain three polyomavirus species. One species, bovine polyomavirus 1 (BoPyV1), was originally discovered as a contaminant in laboratory FCS. A previously unknown species, BoPyV2, occupies the same clade as human Merkel cell polyomavirus and raccoon polyomavirus, both of which are carcinogenic in their native hosts. A third species, BoPyV3, is related to human polyomaviruses 6 and 7. Examples of additional DNA virus families, including herpesviruses, adenoviruses, circoviruses and gyroviruses were also detected either in ground beef samples or in comparison samples of ground pork and ground chicken. The results suggest that the virion enrichment/RCA approach is suitable for random detection of essentially any DNA virus with a detergent-stable capsid. It will be important for future studies to address the possibility that animal viruses commonly found in food might be associated with disease.

## Introduction

In his 2008 Nobel Lecture, Harald zur Hausen noted epidemiological evidence suggesting a correlation between the consumption of beef and the occurrence of colorectal cancer (zur Hausen, 2008, 2012). He speculated that a thermostable virus endemic to cattle might theoretically play a causal role in human colorectal cancer. This conjecture rests on two hypotheses: (1) that one or more tumour viruses are commonly present in beef products and (2) that such viruses are carcinogenic to humans, perhaps through direct non-productive infection of colonic epithelial cells. In this study, we set out to experimentally test the first hypothesis.

Members of the viral family *Polyomaviridae* can survive temperatures typically used for cooking beef (Nims & Plavsic, 2013; Sauerbrei & Wutzler, 2009; zur Hausen, 2012). Some polyomavirus species, such as murine polyomavirus, human Merkel cell polyomavirus (MCV) and raccoon polyomavirus (RacPyV), appear to cause cancer in their natural hosts (Dela Cruz *et al.*, 2013; Feng *et al.*, 2008). Experimental introduction of other polyomaviruses, such as SV40, into non-native host animals can also result in the induction of various types of cancer (Bouvard *et al.*, 2012). Polyomaviruses thus represent plausible candidates for zur Hausen’s conjecture (zur Hausen, 2009, 2012).

Cattle are known to be infected with at least one species of polyomavirus (Parry & Gardner, 1986). The bovine polyomavirus (BoPyV1 or BPyV) is a common contaminant in laboratory FCS and is a standard environmental marker of cattle waste (Rusiñol *et al.*, 2014; Schuurman *et al.*, 1991). It is not known whether BoPyV1 is ever present in food-grade beef products and it is also unknown whether additional polyomavirus species are endemic in cattle.

Our group has recently developed sequence-unbiased methods for detecting polyomaviruses in complex samples (Pastrana *et al.*, 2013a; Schowalter *et al.*, 2010). The methods involve nuclease digestion of non-encapsidated DNA followed by enrichment of virions using Optiprep (iodixanol) velocity/density gradients. The enriched viral DNA is subjected to rolling circle amplification (RCA) using the DNA polymerase of bacteriophage phi29, which geometrically amplifies circular DNA templates using random hexamer primers (reviewed by Johne *et al.*, 2009). The resulting RCA products are suitable for next-generation sequencing (NGS) using the Illumina Nextera/MiSeq platform.

In the USA, industrially produced hamburger meat is often assembled from the trim of many dozens of animals from a wide range of different geographical origins (Drexler, 2002). This could present an ideal situation for virus-hunting, in the sense that testing of only a few specimens might allow the successful detection of infections that affect only a small proportion of the cattle population. We set out to apply our polyomavirus detection techniques to ground meat samples purchased from three large supermarket chains.

## Results

### Deep sequencing of viral DNA-enriched preps from ground meat samples

Meat samples were purchased from three separate supermarkets near Bethesda, Maryland, USA. One supermarket is part of a national chain of stores, while the other two supermarkets belong to Mid-Atlantic regional chains. Ground beef samples 1–3 are samples of store-brand hamburger meat from each of the three supermarkets. Ground beef 4 is a name-brand pre-packaged hamburger patty product advertised as consisting of meat from grass-fed Angus cattle. We suspect (but could not confirm) that ground beef 4 comes from a smaller supply chain and might represent a smaller number of animals. For comparative purposes, samples of store-brand ground chicken, ground pork and a single beef kidney were purchased from regional-chain stores.

The samples were processed for virion enrichment, RCA and NGS (see Methods). Each sample yielded roughly one to two million reads (Table 1). A bioinformatics pipeline was constructed to remove reads with a high degree of homology to the cognate animal genome or to known bacterial species. A few thousand reads with strong homology to plasmids commonly used with our lab’s centrifuge equipment were also bioinformatically subtracted from the read sets.

**Table 1.** Summary of the top three hits for known viruses in each sample. Each sample yielded roughly one to two million total reads. blastn was used to identify reads with high homology to the nucleotide sequences of known viral species found in the NCBI RefSeq database. The percentage coverage indicates the fraction of the viral genome represented in the read set

Sample	Total reads (millions)	Virus 1; no. of reads (coverage)	Virus 2; no. of reads (coverage)	Virus 3; no. of reads (coverage)
Beef kidney	1.1	Bovine adenovirus-6; 294 (33 %)	Coliphage phiX174; 40 (72 %)	Bovine polyomavirus-1; 18 (37 %)
Ground beef 1	1.9	Bovine polyomavirus-1; 69 506 (99 %)	Brochothrix phage NF5; 396 (31 %)	Brochothrix phage BL3; 358 (26 %)
Ground beef 2	1.8	Bovine polyomavirus-1; 486 (95 %)	Bovine herpesvirus-4; 434 (7 %)	Coliphage phiX174 36; (71 %)
Ground beef 3	1.8	Bovine polyomavirus-1; 7758 (99 %)	Brochothrix phage BL3; 58 (12 %)	Brochothrix phage A9; 72 (10 %)
Ground beef 4	2.2	Brochothrix phage A9; 1188 (33 %)	Brochothrix phage NF5; 34 (10 %)	Coliphage phiX174; 50 (80 %)
Ground chicken	1.4	Avian gyrovirus-2; 65, 332 (99 %)	Chicken anaemia virus; 40 294 (97 %)	Gyrovirus-4; 4986 (99 %)
Ground pork	0.8	Torque teno sus virus-1; 1872 (99 %)	Sus scrofa papillomavirus-1; 430 (99 %)	Porcine parvovirus-6; 58 (63 %)

blastn was used to identify reads with high nucleotide homology to known viral species represented in NCBI’s RefSeq database. Bacteriophages tropic for *Brochothrix thermosphacta* (a bacterial species commonly involved in meat spoilage) (Kilcher *et al.*, 2010) or the common enteric bacterium *Escherichia coli* were detected (Table 1). The observed phages are consistent with the expected spectrum of bacterial contamination in food-grade meat products.

Nearly all the samples contained large numbers of reads with homology to at least one DNA virus species known to infect the cognate host animal (Table 1). Most notably, each store-brand ground beef sample contained reads with homology to BoPyV1. Likewise, the ground chicken sample showed reads with homology to chicken gyroviruses and the ground pork sample contained reads with homology to torque teno sus virus-1, Sus scrofa papillomavirus-1, porcine parvovirus-6 and porcine circovirus-2.

Reads that did not show a high degree of nucleotide homology to known targets were subjected to *de novo* contig assembly. blastx was used to search for contigs with distant homology to Large T antigens (LT) or VP1 capsid proteins of various known polyomavirus species. This approach suggested the presence of three additional candidate polyomaviruses in the ground beef samples. Two viruses, which we designated BoPyV2a and BoPyV2b, are >85 % similar to one another and can thus be considered two distinct strains of a single polyomavirus species (Johne *et al.*, 2011). Contigs representing a candidate third bovine polyomavirus species (BoPyV3) were also detected.

A comprehensive search of contigs and individual MiSeq reads revealed only a small handful of additional polyomavirus-like sequences, but none of these additional candidate polyomavirus reads could be confirmed by PCR. The results suggest that if any additional polyomaviruses were present in this set of samples their relative abundance was at least several thousand-fold lower than the detected BoPyVs.

### Confirmation of bovine polyomavirus maps

Back-alignment of the assembled contigs against individual MiSeq reads revealed a variety of apparent polymorphisms within each of the four BoPyVs. Because the MiSeq reaction provides closely paired reads of <300 bp, it is not possible to know which of the polymorphisms were linked to one another on the original ~5 kb viral template DNA molecules. We therefore elected to use PacBio sequencing (which provides very long reads) to validate each candidate BoPyV map. Each candidate BoPyV was targeted with two separate pairs of outward-directed primers to perform whole-genome PCR amplifications using RCA products as templates. The PCR products were subjected to PacBio SMRTbell sequencing. Validated consensus sequences were deposited in GenBank (accession numbers KM496323–6).

Although it seems likely that the map of each PacBio-confirmed virus represents a single viral quasispecies present in the complex swarm of sequences observed in the MiSeq data, a possible caveat is that PCRs can generate chimeric products derived from two or more distinct templates. We attempted to address this caveat by separately analysing two distinct PCR products for each virus, on the assumption that it would be unlikely that the same chimerization event would occur in two separate PCRs.

Additional PCRs were performed to generate plasmids carrying complete BoPyV genomes. In each case, the PCR primers overlapped a unique restriction site in the viral genome, such that the complete viral genome can be reconstituted by excision and intramolecular religation. Two separate BoPyV1 clones showed complex insertions and deletions in the LT antigen introns as well as a wide variety of polymorphisms, some of which were not observed in the PacBio analysis. The BoPyV2b and BoPyV3 clones showed a smaller number of differences relative to the consensus sequences. Surprisingly, separate BoPyV2a clones that used either ground beef 1 or ground beef 3 as a PCR template both showed sequences 100 % identical to the consensus sequence. This is consistent with the much lower number of polymorphisms observed for BoPyV2a in the PacBio read set.

### Phylogenetic analysis of bovine polyomaviruses

**Fig. 1.**
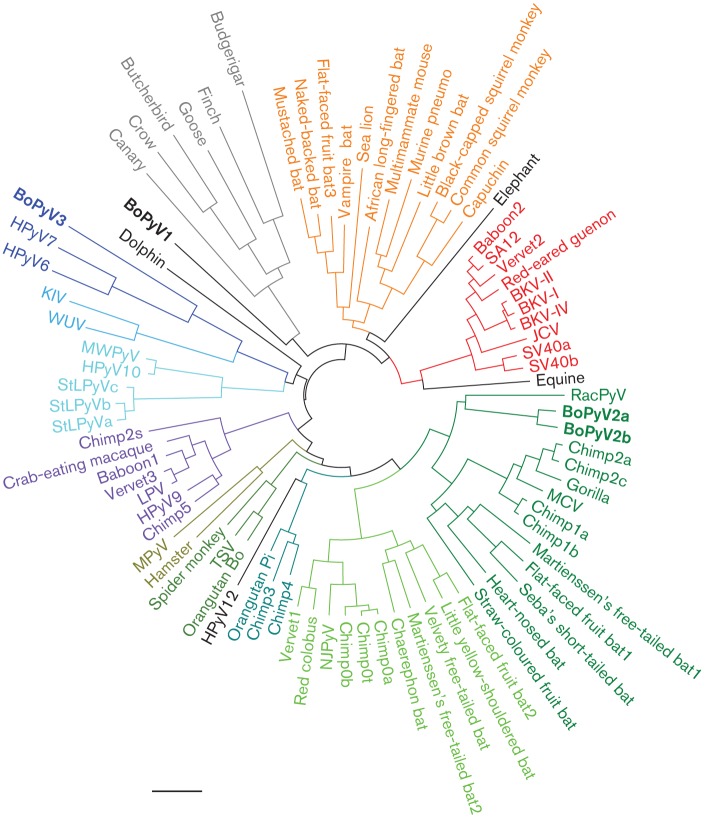
A phylogenetic tree was reconstructed from complete nucleotide sequences of selected polyomavirus genomes (Dereeper *et al.*, 2008). Major clades supported by bootstrap values ≥0.95 have been assigned colours. Species with uncertain clade assignments are shown in black. Polyomavirus species observed in the current study are in bold. See http://home.ccr.cancer.gov/Lco/PyVE.asp for a naming key and accession numbers. Bar, 0.2 substitutions per site.

BoPyV2 occupies a clade that encompasses RacPyV and MCV (Fig. 1). Like other members of the clade, BoPyV2 encodes an overprinted Alternative Large T ORF (ALTO) (Carter *et al.*, 2013) and does not encode a traditional VP3 minor capsid protein (Schowalter & Buck, 2013). Another novel species, BoPyV3, occupies the same clade as human polyomaviruses 6 and 7 (HPyV6 and HPyV7) (Schowalter *et al.*, 2010) and shares key genetic characteristics with these viruses. BoPyV3 is the first non-human member of the broader ‘Wuki’ clade (Johne *et al.*, 2011).

### An attempt to quantify bovine polyomaviruses in ground beef samples

We attempted to use quantitative real-time PCR to estimate the abundance of BoPyV sequences in total DNA extracted from the original meat samples. Although some of the samples sometimes showed signals at cycle thresholds in the mid-30s, the quantification varied considerably between experiments. The results suggest that none of the BoPyVs were present at levels above the threshold of reliable real-time PCR quantification, which we estimate to be roughly two million viral DNA copies per gram of tissue.

The original MiSeq datasets were probed for reads with high nucleotide homology to the confirmed BoPyV maps. Read counts, which presumably roughly reflect the relative abundance of each BoPyV, are shown in Table 2.

**Table 2.** Number of individual reads for each bovine polyomavirus in each sample. Reads observed in ground chicken and pork may represent spillover due to misreading of barcodes in the MiSeq run

Sample	BoPyV1	BoPyV2a	BoPyV2b	BoPyV3
Beef kidney	31	0	9	20
Ground beef 1	71 592	7727	15 998	31 887
Ground beef 2	552	8	1	1158
Ground beef 3	7952	1689	1	0
Ground beef 4	8	0	0	0
Ground chicken	44	2	12	11
Ground pork	37	3	8	8

## Discussion

The primary goal of this study was to search for polyomaviruses in food-grade meat products. Our finding that three (and apparently only three) polyomavirus species are commonly detectable in food-grade ground beef does not provide any indication of whether the presence of viruses in meats has any bearing on the health of humans or cattle. Future studies aimed at experimentally addressing this unanswered question could focus on deep sequencing of tumours, pre-cancerous lesions or other diseased tissues. Sero-epidemiological approaches might also be useful. Older studies have established that, although individuals with occupational exposure to cattle are often seropositive for reactivity against BoPyV1 virions, BoPyV1 seropositivity in the general population is rare (Parry & Gardner, 1986). It is important to note that, in the original zur Hausen conjecture, bovine polyomaviruses might establish a non-productive infection of the colonic epithelium and this type of dead-end infection might not result in the production of the viral capsid proteins. An appropriate screen for virally non-productive exposure to BoPyVs in the general population might examine seroreactivity against the viral tumour antigens (Paulson *et al.*, 2010). In the event that connections are ultimately made between bovine polyomaviruses and disease, it is possible to envision the development of high-potency virus-like particle vaccines (Pastrana *et al.*, 2013b) that could be used to immunize cattle.

It has previously been established that BoPyV1 is a common contaminant in laboratory FCS and that some cultured cell lines are chronically infected with the virus (Howley *et al.*, 1979; Richmond *et al.*, 1984; Waldeck & Sauer, 1977). We have recently begun follow-up studies to address the question of whether BoPyV2, BoPyV3 or other DNA viruses are also present in laboratory FCS. It will be important to consider the question of whether viruses present in FCS might alter the outcomes of some types of cell culture experiments.

Despite the fact that the virion enrichment/RCA approach used in this study was optimized for discovery of polyomaviruses, we inadvertently detected a broad range of eukaryotic DNA virus families. Although phi29 DNA polymerase is particularly effective for geometric amplification of circular DNA molecules, it is also widely used for multiple displacement amplification of long segments of linear DNA. Thus, our detection of an adenovirus and a herpesvirus (both of which have large linear-DNA genomes) alongside circular DNA viral species is not entirely unexpected. Our observations support the idea that the virion enrichment/RCA method is suitable for the robust detection of essentially any DNA virus with a detergent-soluble capsid that is impermeant to nucleases and capable of migrating down an Optiprep ultracentrifuge gradient.

A current model is that polyomaviruses tend to co-evolve with a single host animal species in a relatively uniform fashion (Johne *et al.*, 2011). It is rare to find ~15 % divergence between two polyomavirus subspecies within a given host animal. This level of divergence is instead more typical of polyomaviruses found in closely related host animals (Leendertz *et al.*, 2011; Yamaguchi *et al.*, 2013). A possible explanation for the intermediate level of divergence of the BoPyV2 subspecies might be that one of the subspecies was recently transmitted to cattle from a closely related bovid, such as American bison (with which cattle are sometimes pastured and interbred). A recent transmission bottleneck might explain the surprisingly limited sequence diversity we observed for BoPyV2a. Another conceivable explanation for the unusual level of divergence between BoPyV2a and 2b could be that one of the two polyomavirus subspecies originated from a non-cattle ungulate present in the meat mixture (Veterinary Record, 2014).

While this manuscript was under review, Zhang and colleagues reported a fascinating NGS survey of supermarket meat samples (Zhang *et al.*, 2014). The survey, which used the MiSeq platform to directly sequence all nuclease-resistant nucleic acids (without RCA), detected 31 reads with homology to polyomaviruses in a pool of multiple beef samples purchased from meat markets in San Francisco. PCR amplification of the polyomavirus-like target reads revealed the full genome of BPyV2-SF, which is 99.4 % identical to the BoPyV2b isolate reported in the current study. Zhang and colleagues did not report detection of BoPyV1, 2a or 3. Analysis of read counts suggests that, compared to direct sequencing, our virion enrichment/RCA approach increases the sensitivity of polyomavirus detection by at least a thousand-fold. It will be interesting to see future applications of the enrichment/RCA method to additional types of tissue samples.

## Methods

### 

#### Virion enrichment.

A viral DNA detection approach suitable for use with gram amounts of tissue was adapted from previously reported methods (Pastrana *et al.*, 2013a; Schowalter *et al.*, 2010). Detailed protocols are provided on our lab website http://home.ccr.cancer.gov/Lco/circulome.asp. Roughly 3 g of tissue were minced into fine pieces using razor blades then placed in 15 ml of digest buffer [Dulbecco’s PBS (DPBS) supplemented with 1 mM MgCl_2_, 1 % Triton X-100, 0.02 % Benzonase (Sigma) and 0.3 % Plasmid Safe Exonuclease (Epicentre)] and incubated at 37 °C for 1 h. Several milligrams of collagenase H (Sigma no. C8051) were added and the slurry was mixed and incubated overnight at 4 °C. The digested slurry was buffered to pH ~7 by adding 25 mM ammonium sulfate from a 1 M pH 9 stock and incubated at 37 °C for 30 min. Samples were adjusted to 0.85 M NaCl by addition of 2.5 ml of 5 M NaCl and incubated for 15 min at room temperature, then clarified by centrifugation at 2000 ***g*** for 10 min. The clarified supernatant was reserved. The pelleted material was resuspended in 10 ml of DPBS containing a total of 0.8 M NaCl and 1 % Triton X-100, sonicated in a cup sonicator (Misonix) for 30 s, then recentrifuged at 2000 ***g*** for 10 min at room temperature. The two supernatants were combined and centrifuged onto a 1.5 ml cushion of 39 % iodixanol (Optiprep, Sigma) in DPBS with 0.8 M NaCl at 110 000 ***g*** for 2 h. The bottom ~3 ml (including the Optiprep cushion) were collected and layered onto a 2.3 ml gradient consisting of 27 %, 33 % and 39 % steps of Optiprep in DPBS with 0.8 M NaCl. Gradients were centrifuged in an SW-55 rotor (Beckman) at 234 000 ***g*** for 3.5 h. Fractions were collected by bottom puncture of the tube. A first (bottom) gradient fraction of ~500 µl was collected and discarded. Subsequent fractions of ~200 µl each were retained for analysis.

#### RCA of encapsidated DNA.

DNA was extracted from gradient fractions 3–8 (encompassing roughly the middle third of the gradient, where polyomaviruses are expected to migrate). A 160 µl sample of each fraction was treated with 40 µl of digest buffer [250 mM Tris pH 8, 125 mM EDTA, 2.5 % SDS, 2.5 % proteinase K (Qiagen no. 191331) and 50 mM DTT]. The digestion was incubated at 50 °C for 15 min then at 72 °C for 10 min. DNA was precipitated by addition of 100 µl of 7.5 M ammonium acetate and 2.6 volumes of 95 % ethanol followed by overnight incubation at 4 °C (Crouse & Amorese, 1987). Samples were brought back to room temperature, centrifuged for 1 h at room temperature, washed with 70 % ethanol, briefly air-dried and the pelleted DNA was redissolved in 10 µl of the Sample Buffer supplied with the RCA kit (TempliPhi, GE Healthcare no. 25-6400-50). Samples were heated to 95 °C for 3 min, allowed to cool, then mixed with 10 µl of the instructed Reaction Buffer/Enzyme mix. The RCA reaction was allowed to proceed at 30 °C for 24 h, then the enzyme was heat-inactivated at 65 °C for 10 min. Amplified DNA was ethanol-precipitated and dissolved in 55 µl of 2 mM Tris pH 8, 0.2 mM EDTA.

#### Initial NGS.

RCA samples were prepared for deep sequencing using the Nextera XT DNA sample kit as suggested by the manufacturer (Illumina). A total of 24 samples were barcoded and analysed on the MiSeq platform using a V3 600 cycle cassette. Reads for each sample were assembled into contigs using Trinity software (Grabherr *et al.*, 2011). The list of top viral hits (Table 1) was generated from the alignment of concordant paired reads (with the expected relative mate orientation and within the expected range of distances) against the NCBI RefSeq viral database using Bowtie 2 software (Langmead & Salzberg, 2012). Table 2 was compiled by counting aligned individual reads (regardless of the behaviour of the mate pair) to BoPyV nucleotide templates.

#### Confirmation of BoPyV genome maps.

Polyomavirus genomes are circular and RCA products of circular templates are expected to consist of a long series of tandem repeats. It is thus possible to perform PCR-based amplification of polyomavirus genomes simply by designing outward-directed primers based on known sequence fragments. Outward-directed PCR to amplify nearly full-length polyomavirus genomes was performed using Herculase II fusion DNA polymerase (Agilent). Two separate outward-directed PCRs were performed for each candidate polyomavirus genome. The BoPyV1 and BoPyV3 PCRs used ground beef 1 RCA product as a template, the BoPyV2a and BoPyV2b PCRs used ground beef 3 RCA product as a template. Primers are listed in Table S1 (available in the online Supplementary Material). PCR amplification consisted of 30 cycles of 20 s at 95 °C, 20 s at 52 °C and 3 min at 72 °C. Reactions were performed on an Eppendorf Mastercycler Pro PCR thermal cycler.

The PCR products were used to construct SMRTbell sequencing libraries using Pacific Biosciences DNA Template Prep kit 2.0 (001-540-835) according to the 5 kb Template Preparation and Sequencing protocol, using 1 µg of PCR product as input for each sample. SMRTbell libraries were bound to polymerases using the DNA/Polymerase Binding kit P4 (100-236-500) and Sequencing Primer v2 (001-560-740). Polymerase-bound SMRTbell libraries were linked to Pacific Biosciences MagBeads using the MagBead kit (100-133-600) and were sequenced using a Pacific Biosciences RS II sequencer using C2 chemistry and 180 min movies. One SMRT cell was used for each two pools of PCR products, yielding 82 673 and 69 347 polymerase reads. The PacBio Long Amplicon Analysis tool was used to assemble reads into individual scaffolds and these scaffolds were further validated by back-comparison to the original MiSeq datasets. Analysis of the PacBio results showed that the BoPyV2b primers inadvertently amplified only BoPyV2a PCR products. BoPyV2b polymorphisms were therefore validated only by back-comparison to the original MiSeq reads.

At least one individual whole-genome PCR product for each BoPyV was captured using Gateway technology (Invitrogen) and pFunnyfarm (Schowalter *et al.*, 2010). In all cases, the RCA product of ground beef 1 was used as a template. For BoPyV2a, an additional clone was captured from a PCR that used ground beef 3 as template. Nucleotide maps of plasmids carrying religatable BoPyV genomes are available on our laboratory website http://home.ccr.cancer.gov/Lco/packaging.htm or through https://Addgene.org. The posted maps include annotations of major polymorphisms observed in the NGS datasets.

#### Phylogenetic analysis.

A representative selection of known polyomavirus species and subspecies was downloaded from GenBank. Each polyomavirus species was assigned a nickname based on the common name of the host animal with which it is primarily associated or a familiar abbreviation. A naming key and a curated set of polyomavirus-related sequences are provided on our laboratory website http://home.ccr.cancer.gov/Lco/PyVE.asp. The nucleotide sequence of the complete genome for each virus was compiled and analysed using ‘One Click’ settings (without Gblocks) on the Phylogeny.fr website (Dereeper *et al.*, 2008).

#### Quantitative real-time PCR.

DNA was extracted from 10–15 mg of tissue using the DNeasy Blood & Tissue kit (Qiagen), with a final elution volume of 200 µl, according to the manufacturer’s instructions. One to 5 µl of purified DNA were subjected to a 10–25 µl qPCR using VeriQuest SYBR Green qPCR Master Mix 2X (Affymetrix), as directed by the manufacturer. Reactions were monitored on a 7900HT Fast Real-Time PCR system (Applied Biosystems). Primers used for qPCRs are provided in Table S2. PCR amplicons were generated for each primer pair using RCA products as template. The amplicons were gel-purified, quantified and used as known standards for calculation of copy numbers. The cycle thresholds for the 100 copy standards for each viral target typically ranged from roughly 28 to 32. Since replicates of the 10 copy standard showed poor reproducibility within and among experiments, an arbitrary threshold of 100 copies per reaction was chosen as the limit of detection. Signals were sometimes observed below the 100 copy threshold but these low signals showed poor reproducibility. None of the samples were ever positive above the 100 copy threshold. Since the reactions used DNA template amounts corresponding to ~50–300 µg of tissue, the negative >100 copy results suggests that there were at most two million viral DNA molecules per gram of tissue.
